# Association between kidney function, nutritional status and anthropometric measures in older people: The Screening for CKD among Older People across Europe (SCOPE) study

**DOI:** 10.1186/s12877-020-01699-1

**Published:** 2020-10-02

**Authors:** Agnieszka Guligowska, Andrea Corsonello, Małgorzata Pigłowska, Regina Roller-Wirnsberger, Gerhard Wirnsberger, Johan Ärnlöv, Axel C. Carlsson, Lisanne Tap, Francesco Mattace-Raso, Francesc Formiga, Rafael Moreno-Gonzalez, Ellen Freiberger, Cornel Sieber, Pedro Gil Gregorio, Sara Laínez Martínez, Rada Artzi-Medvedik, Ilan Yehoshua, Paolo Fabbietti, Fabrizia Lattanzio, Tomasz Kostka, on behalf of SCOPE investigators, Fabrizia Lattanzio, Andrea Corsonello, Silvia Bustacchini, Silvia Bolognini, Paola D’Ascoli, Raffaella Moresi, Giuseppina Di Stefano, Cinzia Giammarchi, Anna Rita Bonfigli, Roberta Galeazzi, Federica Lenci, Stefano Della Bella, Enrico Bordoni, Mauro Provinciali, Robertina Giacconi, Cinzia Giuli, Demetrio Postacchini, Sabrina Garasto, Annalisa Cozza, Francesco Guarasci, Sonia D’Alia, Romano Firmani, Moreno Nacciariti, Mirko Di Rosa, Paolo Fabbietti, Gerhard Hubert Wirnsberger, Regina Elisabeth Roller-Wirnsberger, Carolin Herzog, Sonja Lindner, Francesco Mattace-Raso, Lisanne Tap, Gijsbertus Ziere, Jeannette Goudzwaard, Tomasz Kostka, Agnieszka Guligowska, Łukasz Kroc, Bartłomiej K. Sołtysik, Małgorzata Pigłowska, Agnieszka Wójcik, Zuzanna Chrząstek, Natalia Sosowska, Anna Telążka, Joanna Kostka, Elizaveta Fife, Katarzyna Smyj, Kinga Zel, Rada Artzi-Medvedik, Yehudit Melzer, Mark Clarfield, Itshak Melzer, Ilan Yehoshua, Yehudit Melzer, Francesc Formiga, Rafael Moreno-González, Xavier Corbella, Yurema Martínez, Carolina Polo, Josep Maria Cruzado, Pedro Gil Gregorio, Sara Laínez Martínez, Mónica González Alonso, Jose A. Herrero Calvo, Fernando Tornero Molina, Lara Guardado Fuentes, Pamela Carrillo García, María Mombiedro Pérez, Alexandra Renz, Susanne Muck, Stephan Theobaldy, Andreas Bekmann, Revekka Kaltsa, Sabine Britting, Robert Kob, Christian Weingart, Ellen Freiberger, Cornel Sieber, Johan Ärnlöv, Axel Carlsson, Tobias Feldreich

**Affiliations:** 1 Department of Geriatrics, Healthy Ageing Research Centre, Medical University of Lodz, Łódź, Poland; 2 Italian National Research Center on Aging (IRCCS INRCA), Ancona, Fermo and Cosenza, Italy; 3 Laboratory of Geriatric Pharmacoepidemiology and Biostatistics, IRCCS INRCA, Via S. Margherita 5, 60124 Ancona, Italy; 4 Department of Internal Medicine, Medical University of Graz, Graz, Austria; 5 Department of Medical Sciences, Uppsala University, Uppsala, Sweden; 6 School of Health and Social Studies, Dalarna University, Falun, Sweden; 7 Division of Family Medicine, Department of Neurobiology, Care Sciences and Society, Karolinska Institutet, Huddinge, Sweden; 8 Section of Geriatric Medicine, Department of Internal Medicine, Erasmus MC, University Medical Center Rotterdam, Rotterdam, The Netherlands; 9 Geriatric Unit, Internal Medicine Department, Bellvitge University Hospital – IDIBELL – L’Hospitalet de Llobregat, Barcelona, Spain; 10 Department of Internal Medicine-Geriatrics, Institute for Biomedicine of Aging, Krankenhaus Barmherzige Brüder, Friedrich-Alexander Universität Erlangen-Nürnberg, Koberger Strasse 60, 90408 Nuremberg, Germany; 11 Geriatric Department, Hospital Clínico San Carlos, Madrid, Spain; 12 The Recanati School for Community Health Professions at the Faculty of Health Sciences at Ben-Gurion University of the Negev, Beer-sheva, Israel; 13 Maccabi Healthcare Services Southern Region, Tel Aviv-Yafo, Israel

**Keywords:** Aging, Chronic kidney disease, MNA, Malnutrition, Undernutrition, Overweight, Obesity

## Abstract

**Background:**

Different mechanisms connect the nutritional status with the occurrence and the course of chronic kidney disease (CKD). The end-stage renal disease is complicated by catabolic inflammatory reactions and cachexia which leads to malnutrition (undernutrition). On the other hand, obesity is an important risk factor for the development and acceleration of CKD.

**Methods:**

In the SCOPE study, community-dwelling persons aged 75 years and over, from 6 European countries and Israel were examined at the baseline phase. We assessed the relationship between anthropometric measures (Body Mass Index (BMI), circumferences of arm (AC), waist (WC), hip (HC), and calf (CC), waist-to-hip ratio - WHR, waist-to-height ratio - WHtR, risk of malnutrition (Mini Nutritional Assessment - MNA), serum albumin) and estimated glomerular filtration rate (eGFR) calculated by Berlin Initiative Study (BIS) equation.

**Results:**

We studied 2151 subjects (932 men and 1219 women) with a mean age of 79.5 ± 5.9 years. A total of 1333 (62%) participants had CKD (GRF < 60 ml/min/1.73 m^2^). Negative correlations between eGFR and weight, AC, WC, HC, CC, BMI, WHtR were observed. Positive correlation occurred between eGFR and MNA score (Spearman’s rho = 0.11) and albumin concentration (rho = 0.09). Higher weight, AC, WC, HC, CC, BMI and WHtR increased the odds ratio of CKD; higher MNA (OR = 0.98, 95% CI 0.94–1.0) and higher serum albumin (OR = 0.73, 95% CI 0.53–1.0) were weakly associated with reduced odds. The risk of malnutrition was the highest with eGFR < 30 as compared to eGFR > 60 (OR = 2.95, 95%CI = 1.77–4.94 for MNA < 24; OR = 5.54, 95%CI = 1.66–18.5 for hypoalbuminemia < 3.5 g/dL).

**Conclusion:**

The population of community dwelling people aged 75+ with CKD shows general features of overweight and obesity with a small prevalence of malnutrition. For anthropometric measures, the strongest association with eGFR and the highest odds of CKD were identified using WC, HC, CC and WHtR. Albumin level and MNA, but not MNA Short Form, indicated an increased odds of malnutrition with a decrease in eGFR.

## Background

In recent years, an increase in the prevalence of chronic kidney disease (CKD) has been observed [1]. Currently, in the European population, about 18% of citizens suffer from CKD [2]. Similarly, the prevalence of obesity in the worldwide population aged 65 years and older is between 18 and 30% [3, 4]. Not only obesity affects the elderly, but more and more attention is also paid to the occurrence of malnutrition or undernutrition [5]. Depending on the criterion used and setting, it occurs in about 10% among independent people up to more than 60% in rehabilitation hospitals and intensive care units [3].

Nutritional status defined as “the condition of the body in those respects influenced by the diet; the levels of nutrients in the body and the ability of those levels to maintain normal metabolic integrity” [6] contributes to the older people’s health status and risk of many diseases. In older age this status is influenced by the cumulative effect of comorbidities, functional status, medical history and economic status. Hormonal, metabolic and other changes may affect the nutritional status of the elderly, predisposing to deviations from normality.

While the association between CKD and nutritional disorders in the general population is well known [7], there is a substantial lack of evidence with regard to older people (i.e. the population more exposed to the burden of both CKD and malnutrition) [8–10]. CKD may be associated with relevant changes in appetite and taste perception, as well as impaired thirst mechanism and increased risk of dehydration [11]. All these changes may increase the risk of malnutrition [12], which in turn may lead to functional impairment [13, 14], sarcopenia and frailty [15], increased risk of falls [16], hospitalization and mortality [17, 18]. On the other hand, age-related hormonal changes together with lower physical activity may promote obesity in the older adults [19, 20]. Excessive body fat, especially visceral, is associated with metabolic disorders and increased inflammation [21], CKD, hypertension and diabetes [22, 23].

In the Screening for Chronic Kidney Disease Among Older People Across Europe (SCOPE) project a lot of attention has been paid to the assessment of participants’ nutritional status [24]. The collected data allows reliable and detailed analysis of the relationship between kidney function and the nutritional status. The aim of our study was to assess the relationship between anthropometric measures, malnutrition and kidney function among older adults aged 75 or more. Given that appropriate management of older patients with CKD may contribute to slower loss of kidney function (potentially preventing kidney failure) and enable to control better over its consequences [25, 26], understanding the cross-sectional interactions between anthropometric measures, nutritional status and CKD represents a relevant step in prevention strategies.

## Methods

### Design of the study and study population

The SCOPE study (European Grant Agreement no. 436849), is a multicenter prospective cohort study involving patients older than 75 years attending geriatric and nephrology outpatient services in participating institutions in Austria, Germany, Israel, Italy, the Netherlands, Poland and Spain. Only people aged 75 or more were asked to participate because of the high prevalence of CKD in this population [8, 27]. Methods of the SCOPE study have been extensively described elsewhere [24]. Briefly, all patients attending the outpatient services at participating centers from August 2016 to August 2018 were asked to participate. Only patients signing a written informed consent entered the study. Age greater or equal to 75 years was the only inclusion criteria, the exclusion criteria were: end-stage renal disease or dialysis at time of enrollment; history of solid organ or bone marrow transplantation; active malignancy within 24 months prior to screening or metastatic cancer; life expectancy less than 6 months (based on the judgment of the study physician after careful medical history collection and diagnoses emerging from examination of clinical documentation exhibited); severe cognitive impairment (Mini Mental State Examination < 10); any medical or other reason (e.g. known or suspected patients’ inability to comply with the protocol procedure) in the judgement of the investigators, that the patient was unsuitable for the study; unwilling to provide consent and limited possibility to attend follow-up visits. Enrolled patients underwent an extensive assessment including: demographic data, socioeconomic status, physical examination, comprehensive geriatric assessment, bioimpedance analysis, diagnoses (clinical history and assessment of clinical documentation exhibited by patients and/or caregivers), quality of life, physical performance, overall comorbidity and blood and urine sampling. Patients were followed-up for 24-months as previously described [24]. The study protocol was approved by ethics committees at all participating institutions, and complies with the Declaration of Helsinki and Good Clinical Practice Guidelines. The study was registered at ClinicalTrials.gov (NCT02691546). Only baseline data was used in the present study.

Overall, 2461 patients were initially enrolled in the study, 310 participants were excluded because of missing data in any of the study variables, leaving a final sample of 2151 participants to be included in the analyses.

### Study variables

Each participating center used standardized measurement methods in accordance with the study protocol. Age, gender and country were considered in the analysis.

#### Anthropometric measurements

Trained staff measured participants’ height and weight using a weighing scale with height rod. Waist, hip, arm and calf circumference were measured with a flexible measuring tape to the nearest 0.1 cm according to the WHO’s standard technique. Then, the Body Mass Index (BMI) was calculated by dividing the subject’s weight in kilograms by height in metres squared, Waist-to-Height Ratio (WHtR) as WC divided by height and Waist-to-Hip Ratio (WHR) as quotient of the circumference of the waist to the hips.

#### Risk of malnutrition (undernutrition)

Nutritional status was assessed using the Mini Nutritional Assessment (MNA) questionnaire. This is simple and practical, non-invasive tool which is commonly used by health professionals for early detection of risk of malnutrition [28]. The MNA consists of 18 questions: 5 in the screening part and 13 in the assessment part. The total score ranges from 0 to 30, where a score of less than 17 points indicates malnutrition, from 17 to 23.5 risk of malnutrition, and 24 or more points normal nutrition. The MNA is recommended by the ESPEN for detecting the presence of malnutrition and the risk of developing malnutrition among older adults [3].

#### Biochemical markers of nutritional status

Serum albumin was determined with the use of the bromocresol green colorimetric assay and presented as g/dl [29]. Albumin is a serum hepatic protein with half-life of 14–20 days. Serum albumin level suggesting malnutrition was set at < 3.5 g/dL (hypoalbuminemia) [30].

#### Kidney function

Kidney function was measured, according to estimated glomerular filtration rate (eGFR) category, based on the GFR BIS equation, which is among the few equations specifically developed and validated among people aged 70 or more [31, 32]:

$$ 3736\times {\mathrm{creatinine}}^{\hbox{-} 0.87}\times {\mathrm{age}}^{\hbox{-} 0.95}\left[\times 0.82\ \mathrm{if}\ \mathrm{female}\right] $$

Participants were divided into 4 groups presenting stages of CKD: G1–2 (60 or more); G3A (45–59.9); G3B (30–44.9); G4 (15–30), and were also dichotomized as CKD(−) group (GFR ≥ 60) and CKD(+) group (GRF < 60) [33].

#### Overall comorbidity

Overall comorbidity was assessed by Cumulative Illness Rating Score for Geriatrics (CIRS-G) [34]. CIRS-G consists of the assessment of the severity of coexisting diseases in 14 organ/systems scales, each ranging from 0 (problem absent) to 4 (severe problem with requirement of immediate treatment and/or severe organ/system failure).

#### Diagnoses

Diabetes, cancer, hypertension, coronary artery disease (CAD), cerebrovascular diseases (CVD), heart failure (HF), chronic obstructive pulmonary disease (COPD), and metabolic syndrome were also considered in the analyses. The occurrence of selected diagnoses was ascertained by the study physician through detailed collection of medical history and examination of clinical documentation exhibited by patients and caregivers during study visit as described above. Metabolic syndrome was defined on the basis of the International Diabetes Federation criteria as central obesity (based on WC) plus any two of the following risk factors: plasma triglycerides ≥150 mg/dl, HDL-cholesterol< 40 for men and < 50 for women, blood pressure > 130/80 mmHg or previous diagnosis of hypertension, fasting plasma glucose> 100 mg/dl or previous diagnosis of diabetes [35].

### Statistical analysis

All variables were checked for normality of distribution by the Kolmogorov–Smirnov test. All the continuous variables were not normally distributed, therefore they were presented by median and interquartile difference. Mann Whitney U-test was used to compare nutritional status between CKD(+) and CKD(−) groups and between men and women. Differences between subgroups, depending on CKD stages, were assessed using the Kruskal-Wallis and post hoc Dunn-Sidak test. Spearman correlations between nutritional status indicators and kidney function (eGFR) were calculated. Additionally, test of homogeneity of the slopes (comparison of regression lines and ANOVA interactions) were used to corroborate the different relationship of eGFR to nutritional components according to sex.

Logistic regression (Odds ratios and corresponding 95% confidence intervals (95%CI)) was used to assess which independent variables predicted the presence of CKD (Model 1), malnutrition defined with MNA < 24 (Model 2) and malnutrition defined with albumin < 3.5 g/dL (Model 3). Each model was adjusted for age and sex (A), age, sex, concomitant diseases (diabetes mellitus, cancer, HF, CAD, CVD, COPD, metabolic syndrome) and country (B).

Statistical significance was set at *p* <  0.05. All statistical analyses were performed with SPSS version 24 (SPSS Inc., Chicago, IL, USA).

## Results

We studied 2151 subjects (932 men) with median age 79.5 ± 5.9 years. A total of 1333 (62%) participants had CKD (GRF < 60 ml/min/1.73 m^2^). Table 1 shows the general characteristics of the participants divided by sex and presence of CKD.

**Table 1:** General characteristics of the study population (*n* = 2151) according to sex and prevalence of CKD. The quantitative values are expressed by median and interquartile difference, qualitative values as number and percentage

Variable	All ***n*** = 2151	Men ***n*** = 932	Women ***n*** = 1219	CKD(−) group ***n*** = 818	CKD(+) group ***n*** = 1333
Age (years)	79.5 (5.9)	79.5 (5.8)	79.5 (5.9)	78.6 (4.5)	80.2 (6.2) ^c^
Men (n, %)	932 (43.3)	–	–	315 (38.5)	617 (46.3) ^c^
Education (years)	11 (7)	12 (7)	11 (7) ^a^	12 (8)	11 (6) ^c^
Weight (kg)	72.0 (17.5)	78 (15.6)	67 (16.3) ^c^	70 (18.4)	74 (18.9) ^c^
Arm circumference (cm)	28 (4.0)	28 (3.5)	28 (5.0)	28 (4.0)	28 (4.0) ^c^
Waist circumference (cm)	98 (15)	102 (14)	95 (16) ^c^	96 (15)	99 (16) ^c^
Hip circumference (cm)	104 (11.0)	104 (9.0)	104 (14.0) ^a^	102 (10.1)	105 (12.0) ^c^
Calf circumference (cm)	36 (5.0)	37 (5.0)	35 (5.0) ^c^	36 (5.0)	36 (6.0) ^c^
BMI (kg/m^2^)	27.3 (5.6)	27.2 (5.0)	27.4 (6.3)	26.7 (5.4)	27.6 (5.7) ^c^
WHR	0.93 (0.11)	0.98 (0.09)	0.89 (0.09) ^c^	0.92 (0.12)	0.93 (0.11) ^c^
WHtR	0.60 (0.10)	0.60 (0.09)	0.60 (0.10)	0.59 (0.10)	0.61 (0.09) ^c^
MNA SF	13 (2)	14 (2)	13 (2) ^c^	13 (2)	13 (2)
MNA	27.0 (3.0)	27.0 (2.5)	26.5 (3.0) ^c^	27.0 (2.5)	26.5 (3.0) ^c^
Albumin (g/dL)	4.22 (0.5)	4.30 (0.5)	4.20 (0.4)	4.30 (0.4)	4.20 (0.4) ^c^
Diabetes (n,%)	532 (24.7)	279 (29.9)	253 (20.8) ^c^	154 (18.8)	378 (28.4) ^c^
Cancer (n,%)	368 (17.1)	188 (20.2)	180 (14.8) ^c^	116 (14.2)	252 (18.9) ^b^
Hypertension (n,%)	1635 (76.0)	707 (75.9)	928 (76.1)	542 (66.3)	1093 (82.0) ^c^
CAD (n,%)	288 (13.4)	171 (18.3)	117 (9.6) ^c^	72 (8.8)	216 (16.2) ^c^
CVD (n,%)	268 (12.5)	132 (14.2)	136 (11.2) ^c^	87 (10.6)	181 (13.6) ^a^
HF (n,%)	335 (15.6)	167 (17.9)	168 (13.8) ^b^	68 (8.3)	267 (20.0) ^c^
COPD (n,%)	252 (11.7)	156 (16.7)	96 (7.9) ^c^	70 (8.6)	182 (13.7) ^b^
Metabolic syndrome (n,%)	1257 (58.4)	555 (59.5)	702 (57.6)	403 (49.3)	854 (64.1) ^c^
CIRS-G	8 (6)	8 (7)	7 (7) ^c^	7 (6)	9 (7) ^c^
Country					
Austria (n, %)	225 (10.5)	117 (12.6)	108 (8.9) ^b^	30 (3.7)	195 (14.6) ^c^
Germany (n, %)	270 (12.6)	83 (8.9)	187 (15.3) ^c^	106 (13.0)	164 (12.3)
Israel (n, %)	299 (13.9)	138 (14.8)	161 (13.2)	149 (18.2)	150 (11.3) ^c^
Italy (n, %)	436 (20.3)	221 (23.7)	215 (17.6) ^c^	155 (18.9)	281 (21.1)
The Netherlands (n, %)	285 (13.2)	160 (17.2)	125 (10.3) ^c^	90 (11.0)	195 (14.6) ^a^
Poland (n, %)	352 (16.4)	101 (10.8)	251 (20.6) ^c^	160 (19.6)	192 (14.4) ^b^
Spain (n, %)	284 (13.2)	112 (12.0)	172 (14.1)	128 (15.6)	156 (11.7) ^b^

^a^ *p* <  0.05; ^b^ *p* <  0.01; ^c^ *p* <  0.001

Women showed lower educational level than men, had lower prevalence of diabetes mellitus, cancer, CAD, CVD, HF, COPD, and lower CIRS-G score. Men had higher weight, WHR, AC, WC, HC, CC and higher MNA SF and MNA scores. Women were more frequent among patients enrolled in Germany and Poland, while people from Austria, Italy and the Netherlands were more frequently men (Table 1).

Participants in the CKD(+) group were older, more frequently men, less educated and with a higher prevalence of chronic diseases, including metabolic syndrome. The nutritional status of subjects with CKD was characterized by higher BMI, WHR, WHtR, weight and AC, WC, HC and CC. The result obtained in the MNA SF test did not differ between the groups, while the MNA and albumin were significantly lower in the CKD(+) group compared to patients without CKD. Finally, subjects with CKD were more frequent among patients enrolled in Austria and the Netherlands, while less frequent among those enrolled in Israel, Poland and Spain (Table 1).

Analysis of study variables across eGFR stages 1–4 by sex (Table 2) showed that age increased and education decreased together with reducing eGFR. Higher values of anthropometric measures were observed among patients with more advanced CKD stages, while MNA and albumin level decreased together with reducing eGFR. Likewise, the prevalence of concomitant chronic diseases and CIRS-G continually increased together with eGFR stage. Finally, more advanced stages of CKD were especially prevalent among patients from Austria and the Netherlands, while less frequent in Israel, Italy and Poland (Table 2).

**Table 2:** General characteristics of men (*n* = 931) and women (*n* = 1217) according to eGFR category. The quantitative values are expressed by median and interquartile difference, qualitative values as number and percentage

Variable	Men					Women				
	G1–2 (≥60) ***N*** = 315	G3A (45–59.9) ***N*** = 368	G3B (30–44.9) ***N*** = 198	G4 (15–30) ***N*** = 50	***p***-value*	G1–2 (≥60) ***N*** = 503	G3A (45–59.9) ***N*** = 483	G3B (30–44.9) ***N*** = 193	G4 (15–30) ***N*** = 38	***p***-value*
**Age (years)**	78.7 (4.6)	79.6 (6.0)	80.1 (6.0)	83.2 (7.3)	< 0.001^abcdef^	78.6 (4.4)	79.7 (5.7)	83.1 (7.9)	81.9 (6.8)	< 0.001 ^abcdef^
**Education (years)**	12 (8)	12 (7)	11 (6)	9 (5)	0.031 ^bcdef^	12 (7)	11 (6)	10 (5)	8 (3)	< 0.001 ^abcdef^
**Weight (kg)**	77.0 (14.3)	78.0 (17.0)	81.4 (16.2)	75.5 (18.0)	< 0.001 ^abcdef^	65.0 (16.0)	67.0 (18.0)	69.4 (19.6)	72.7 (16.6)	< 0.001 ^abcdef^
**Arm circumference (cm)**	28 (3.8)	28 (3.0)	29 (3.0)	28 (5.0)	< 0.001 ^bdf^	28 (4.5)	28 (4.5)	28 (5.0)	28 (5.0)	< 0.001 ^bcde^
**Waist circumference (cm)**	100 (14.0)	101 (13.9)	104 (13.0)	102 (14.8)	< 0.001 ^bdf^	92 (17.0)	95 (16.0)	98 (16.0)	102 (13.2)	< 0.001 ^abcdef^
**Hip circumference (cm)**	102 (9.0)	103 (9.0)	106 (9.0)	105 (10.0)	< 0.001 ^bcde^	102 (12.0)	105 (13.4)	108 (15.0)	111 (11.0)	< 0.001 ^abcdef^
**Calf circumference (cm)**	36 (4)	36 (5)	38 (7)	42 (8)	< 0.001 ^bcdef^	35 (4)	36 (5)	36 (6)	41 (13)	< 0.001 ^abcef^
**BMI (kg/m**^**2**^**)**	26.9 (4.5)	27.2 (5.0)	27.7 (4.9)	26.7 (4.7)	0.011 ^abcef^	26.6 (5.8)	27.6 (6.0)	28.5 (7.0)	29.6 (7.5)	< 0.001 ^abcdef^
**WHR**	1 (0.1)	1 (0.1)	1 (0.1)	1 (0.1)	ns	0.89 (0.1)	0.89 (0.1)	0.89 (0.1)	0.90 (0.1)	ns
**WHtR**	0.59 (0.1)	0.60 (0.1)	0.61 (0.1)	0.60 (0.1)	< 0.001 ^bcdef^	0.6 (0.1)	0.6 (0.1)	0.6 (0.1)	0.7 (0.1)	< 0.001 ^cef^
**MNA SF**	14 (2)	14 (1)	14 (2)	14 (3)	ns	13 (2)	13 (2)	13 (2)	13 (3)	ns
**MNA**	27 (2.5)	27 (2.5)	27 (2.5)	26 (4.7)	< 0.001 ^cef^	27 (3.0)	26.5 (3.5)	26 (3.0)	25.0 (6.2)	< 0.001 ^c^
**Albumin (g/dL)**	4.3 (0.4)	4.3 (0.4)	4.3 (0.4)	4.0 (0.5)	< 0.001 ^cef^	4.3 (0.4)	4.2 (0.5)	4.2 (0.5)	4.0 (0.6)	< 0.001 ^c^
**Diabetes (n, %)**	75 (23.8)	104 (28.3)	83 (41.9)	17 (34.0)	< 0.001 ^abcdef^	79 (15.7)	88 (18.2)	70 (36.3)	16 (42.1)	< 0.001 ^abcdef^
**Cancer (n, %)**	51 (16.2)	76 (20.7)	49 (24.7)	12 (24.0)	ns	65 (12.9)	78 (16.1)	29 (15.0)	6 (15.8)	ns
**Hypertension (n, %)**	201 (63.8)	281 (76.4)	177 (89.4)	47 (94.0)	< 0.001 ^abcdef^	341 (67.8)	378 (78.3)	169 (87.6)	38 (100.0)	< 0.001 ^abcdef^
**CAD (n, %)**	36 (21.2)	68 (40.0)	56 (32.9)	10 (5.9)	< 0.001 ^abcdef^	36 (7.2)	36 (7.5)	36 (18.7)	9 (23.7)	< 0.001 ^bcdef^
**CVD (n, %)**	39 (12.4)	57 (15.5)	30 (15.2)	6 (12.0)	ns	48 (9.5)	49 (10.1)	30 (15.5)	9 (23.7)	0.009 ^bcdef^
**CHF (n, %)**	26 (8.3)	74 (20.1)	51 (25.8)	15 (30.0)	< 0.001 ^abcdef^	42 (8.3)	75 (15.5)	40 (20.7)	11 (28.9)	< 0.001 ^abcdef^
**COPD (n, %)**	40 (12.7)	59 (16.0)	44 (22.2)	13 (26.0)	0.011 ^abcdef^	30 (6.0)	36 (7.5)	25 (13.0)	5 (13.2)	0.012 ^abcdef^
**Metabolic syndrome (n, %)**	161 (51.1)	213 (57.9)	145 (73.2)	36 (72.0)	< 0.001 ^abcdef^	242 (48.1)	289 (59.8)	140 (72.5)	30 (78.9)	< 0.001 ^abcdef^
**CIRS-G**	7 (6)	8 (6)	10 (8)	12 (7)	< 0.001 ^abcdef^	6 (5)	7 (6)	11 (7)	12 (6)	< 0.001 ^abcde^
**Country**										
**Austria (n, %)**	12 (3.8)	34 (9.2)	44 (22.2)	26 (52.0)	< 0.001 ^abcdef^	18 (3.6)	37 (7.7)	34 (17.6)	18 (47.4)	< 0.001 ^abcdef^
**Germany (n, %)**	32 (10.2)	35 (9.5)	12 (6.1)	4 (8.0)	ns	74 (14.7)	69 (14.3)	39 (20.2)	5 (13.2)	ns
**Israel (n, %)**	64 (20.3)	54 (14.7)	17 (8.6)	3 (6.0)	0.001 ^abcde^	85 (16.9)	58 (12.0)	16 (8.3)	1 (2.6)	0.002 ^abcdef^
**Italy (n, %)**	73 (23.2)	101 (27.4)	44 (22.2)	3 (6.0)	0.008 ^acdef^	82 (16.3)	107 (22.2)	23 (11.9)	3 (7.9)	0.003 ^abcdef^
**The Netherlands (n, %)**	43 (13.7)	61 (16.6)	46 (23.2)	10 (20.0)	0.042 ^abcdef^	47 (9.3)	40 (8.3)	32 (16.6)	6 (15.8)	0.008 ^cde^
**Poland (n, %)**	49 (15.6)	42 (11.4)	10 (5.1)	0 (0.0)	< 0.001 ^abcdef^	111 (22.1)	115 (23.8)	25 (13.0)	0 (0.0)	< 0.001 ^bcdef^
**Spain (n, %)**	42 (13.3)	41 (11.1)	25 (12.6)	4 (8.0)	ns	86 (17.1)	57 (11.8)	24 (12.4)	5 (13.2)	ns

*p-values = based on comparisons between subgroups

^a^ G1–2 (60 or more) vs G3A (45–59.9)

^b^ G1–2 (60 or more) vs G3B (30–44.9)

^c^ G1–2 (60 or more) vs G4 (15–30)

^d^ G3A (45–59.9) vs G3B (30–44.9)

^e^ G3A (45–59.9) vs G4 (15–30)

^f^ G3B (30–44.9) vs G4 (15–30)

The correlations of eGFR with selected variables are presented in Table 3. Negative correlations with age and positive correlations with education level were found. Negative correlations for all the nutritional anthropometric variables (except for WHR - eGFR relationship in men) with kidney function were observed. A positive correlation occurred between eGFR and MNA score and albumin concentration (Table 3).

**Table 3:** Spearmans correlations between nutritional status indicators and eGFR

Variable	Correlation with GFR All	Correlation with GFR Men	Correlation with GFR Women
Age (years)	−0.274 ^b^	−0.257 ^b^	−0.286 ^b^
Education (years)	0.106 ^b^	0.066 ^b^	0.150 ^b^
Weight (kg)	−0.164 ^b^	−0.098 ^b^	−0.152 ^b^
Arm circumference (cm)	−0.096 ^b^	−0.113 ^b^	− 0.086 ^b^
Waist circumference (cm)	− 0.186 ^b^	− 0.150 ^b^	− 0.160 ^b^
Hip circumference (cm)	− 0.168 ^b^	− 0.154 ^b^	− 0.189 ^b^
Calf circumference (cm)	− 0.167 ^b^	− 0.188 ^b^	− 0.131^b^
BMI (kg/m^2^)	− 0.118 ^b^	− 0.073 ^a^	−0.153 ^b^
WHR	−0.100 ^b^	−0.053	− 0.045 ^a^
WHtR	−0.135 ^b^	− 0.122 ^b^	−0.145 ^b^
MNA SF	0.018	0.023	0.031
MNA	0.111 ^b^	0.102 ^b^	0.134 ^b^
Albumin (g/dL)	0.092 ^b^	0.103 ^b^	0.094 ^b^

^a^ *p* < 0.05; ^b^ *p* < 0.01

**Fig. 1:**
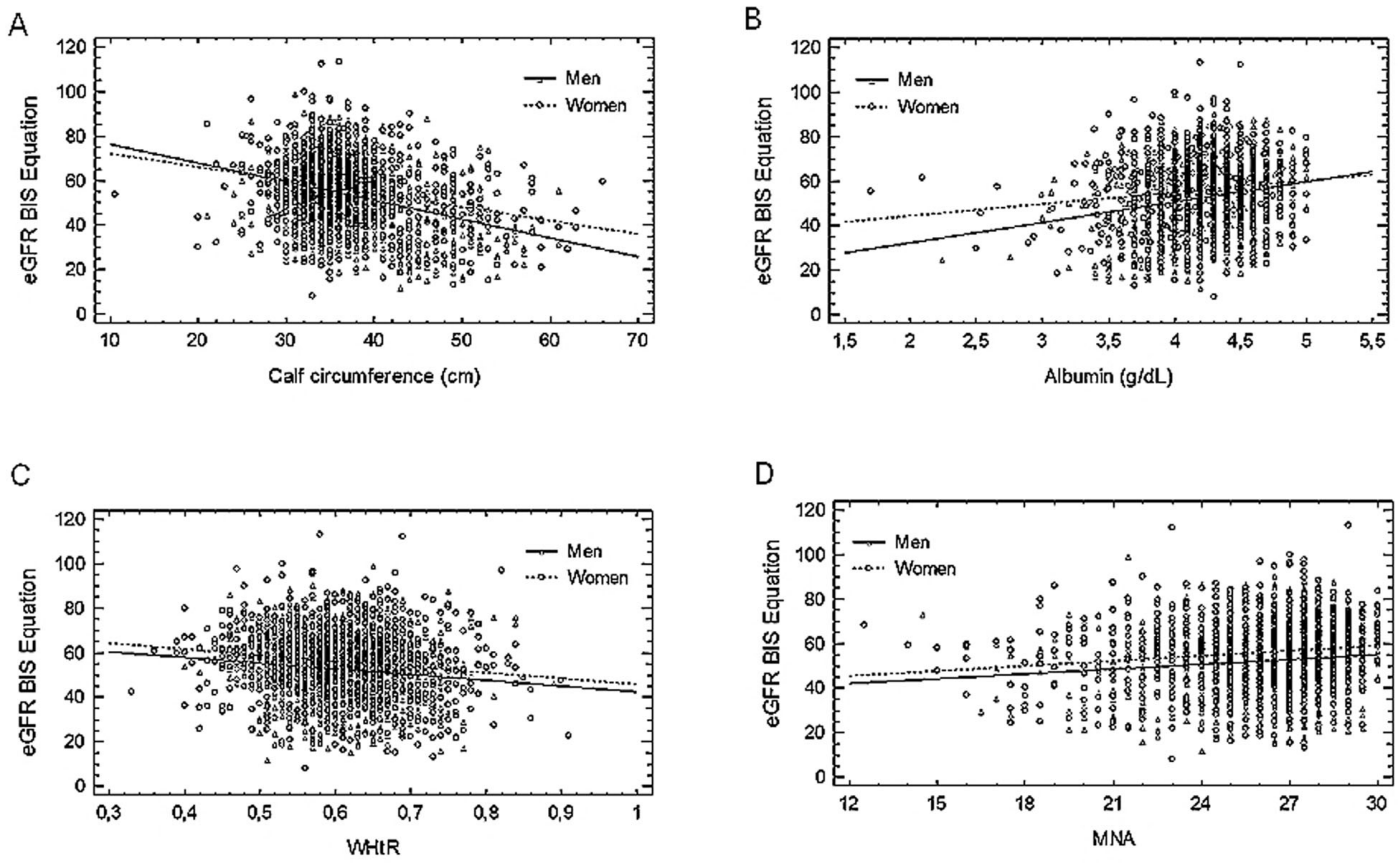
Correlation between eGFR and **a** calf circumference, **b** albumin **c** waist-to-height ratio, **d** Mini Nutritional Assessment. **a** calf circumference rho = −0.188 for men, and rho = − 0.131 for women, *p* < 0.01; **b** albumin rho = 0.103 for men, and rho = 0.094 for women, *p* < 0.01; **c** waist-to-height ratio (WHtR) rho = − 0.122 for men, and rho = − 0.145 for women, *p* < 0.01; **d** Mini Nutritional Assessment (MNA) rho = 0.102 for men, and rho = 0.134 for women, *p* < 0.01

Figure 1a-d shows the relationship of eGFR to selected nutritional variables. Comparison of the slopes of regression lines showed significant differences for eGFR association to CC (Fig. 1a) and albumin levels (Fig. 1b) between men and women. In men, those associations were steeper than in women. For other nutritional variables, those associations were similar in both sexes. As an example, the relationship of eGFR to WHtR has been shown in Fig. 1c and that to MNA in Fig. 1d.

Age and sex adjusted regression models showed an increased risk of CKD (defined as eGFR < 60) for higher weight, AC, WC, HC, CC, BMI and WHtR, as well as a decrease in the risk of CKD with higher MNA and higher serum albumin. After further adjusting for comorbidities and country results remained substantially unchanged (Table 4).

**Table 4:** Logistic regression models (Odds ratios and corresponding 95% confidence intervals (95%CI)) for the association of CKD and malnutrition: the presence of CKD (Model 1) predicted by nutritional and anthropometric variables, while malnutrition defined with MNA < 24 (Model 2) and malnutrition defined with albumin < 3.5 g/dL (Model 3) predicted by CKD stages. Each model was adjusted for age and sex (A), age, sex, concomitant diseases (diabetes mellitus, cancer, HF, CAD, CVD, COPD, metabolic syndrome) and country (B)

**Variables**	**Model 1A** CKD GRF < 60 ml/min/1.73 m^2^		**Model 1B** CKD GRF < 60 ml/min/1.73 m^2^	
	OR (95%CI) age and sex adjusted		OR (95%CI) age, sex, comorbidities^a^ and country adjusted	
Weight (kg)	1.03 (1.02–1.04)		1.02 (1.01–1.03)	
Arm Circumference (cm)	1.10 (1.07–1.13)		1.07 (1.04–1.10)	
Waist circumference (cm)	1.03 (1.02–1.03)		1.01 (1.00–1.02)	
Hip circumference (cm)	1.03 (1.02–1.04)		1.02 (1.01–1.03)	
Calf circumference (cm)	1.08 (1.06–1.10)		1.03 (1.00–1.06)	
BMI (kg/m^2^)	1.07 (1.05–1.09)		1.05 (1.02–1.07)	
WHR	1.10 (0.97–1.25)		0.99 (0.86–1.14)	
WHtR	1.38 (1.22–1.56)		1.18 (1.01–1.38)	
MNA	0.95 (0.92–0.99)		0.98 (0.94–1.00)	
Albumin (g/dL)	0.73 (0.56–0.95)		0.73 (0.53–1.00)	
**Variables** CKD stages GFR in ml/min/1.73 m^2^	**Model 2A** malnutrition MNA < 24	**Model 2B** malnutrition MNA < 24	**Model 3A** malnutrition albumin < 3.5	**Model 3B** malnutrition albumin < 3.5
	OR (95%CI) age and sex adjusted	OR (95%CI) age, sex, comorbidities^a^ and country adjusted	OR (95%CI) age and sex adjusted	OR (95%CI) age, sex, comorbidities^a^ and country adjusted
CKD ≥60	1	1	1	1
CKD 45–59.9	1.12 (0.86–1.45)	1.03 (0.78–1.37)	2.05 (1.02–4.10)	1.70 (0.78–3.72)
CKD 30–44.9	1.51 (1.14–2.00)	1.28 (0.93–1.75)	2.21 (1.20–4.06)	1.71 (0.81–3.61)
CKD 15–30	3.15 (1.98–4.99)	2.95 (1.77–4.94)	3.70 (1.57–8.67)	5.54 (1.66–18.51)

^a^ comorbidities: diabetes, cancer, HF, CAD, CVD, COPD, metabolic syndrome

Finally, a graded association between CKD stage and malnutrition defined with MNA < 24 or hypoalbuminemia was found in age- and sex-adjusted model. After further adjustment for comorbidities and country, only eGFR< 30 remained statistically associated with MNA < 24 or hypoalbuminemia (Table 4).

## Discussion

To the best of our knowledge, this is the first study to analyze the relationship between nutritional status and kidney function in community dwelling people aged 75+ not on dialysis and without ESRD. The results show a clear association between nutritional status and eGFR in this population. Findings about anthropometric measures generally point out that the severity of overweight and obesity is related to the prevalence of CKD. Albumin and MNA, but not MNA SF, correlate with kidney function and discriminate eGFR categories.

As other studies show [36], CKD is primarily associated with abdominal obesity. This type of obesity is characterized by an increase of adipose tissue surrounding the intra-abdominal organs. This phenotype is associated with metabolic disturbances and several chronic diseases [37, 38]. One of them is metabolic syndrome. There is growing evidence that metabolic syndrome is significantly associated with the risk of rapid eGFR decline [39, 40], and its high prevalence among people with CKD in our study suggests that this notion may also apply to older populations. The easiest method to assess the occurrence of this type of obesity is the WC [41]. Central obesity was defined based on cut-off points advocated by Lean et al. (1995) as a WC of ≥102 cm in men, and ≥ 88 cm in women [42]. Such WC are one of the risk factors of metabolic syndrome, hypertension, diabetes, which indirectly affects kidney function. In our study, WC was strongly associated with CKD. Similarly to the study of Evans et al., where researchers emphasize that abdominal obesity is even more strongly associated with CKD risk than BMI [43]. Although BMI is a controversial indicator and there is ongoing discussion regarding the cut-off points for the older adults [44], present analysis showed that the occurrence of kidney disease is associated with higher BMI.

WHtR was another indicator that showed a link between CKD and obesity. This indicator is a universal measure that can be used regardless of sex and race with a cut-off value of WHtR = 0.5. The median for the CKD (+) group was high (0.6 ± 0.1), and reached the highest value of 0.7 ± 0.1 in the group of women in CKD stage G4. In the meta-analyses done by Lee et al. [45] and Correa et al. [46], WHtR was the best discriminator for the risk of hypertension, diabetes, and dyslipidemia in both sexes. WHtR can be simpler and more predictive indicator of the cardiometabolic risk factors associated with central obesity than other anthropometric indices [47, 48]. In the present study WHtR showed the highest odds ratio for the prediction of CKD. The increase of WHtR by 0.1 increased the risk of kidney disease by 38%, and by 18% after adjustment for comorbidities. This easy-to-count indicator can be recommended as a CKD risk screening tool.

In contrast, WHR, as a body build indicator can only be analysed separately for sexes and only for participants categorized as obese in order to assess obesity type [49, 50]. The lack of statistically significant correlation between eGFR and WHR in men and lack of statistical significance in logistic regression analyses seems to suggest minor relevance of this measure in older population.

Non-dominant middle arm circumference constitutes a useful tool as a marker of malnutrition [51]. Our analysis showed a negative correlation with eGFR but it presented a small differentiation between eGFR categories (about 1 cm). Additionally, AC may be loaded with one of the highest anthropometric measurement errors [52]. Therefore, inference based on this result should be limited in older population.

In contrast, the CC showed higher differentiation between eGFR categories. Differences found between subgroups according to the eGFR category, the highest statistically significant correlation coefficients with eGFR and statistical significance in logistic regression analyses indicate a strong relationship between CC and kidney function.

Men with eGFR 15–30 had lower BMI, WHtR and WC as compared to the group of men with eGFR 30–44.9, while the CC was definitely higher. This may be due to the presence of oedema of the lower limbs that accompanies advanced CKD. The lowest values of albumin observed in this group may also partly account for this apparent discrepancy [53, 54]. In women, albumin levels also decrease what is accompanied by an increase in CC but at the same time weight, BMI and WHtR were also higher. A detailed analysis of body composition should clarify those potential sex differences.

Albumin level and MNA were used as markers of nutritional status and malnutrition. Decrease in albumin level may be associated with a decrease in renal function and loss of albumin in the urine. This phenomenon is escalating with the progression of CKD [55]. Regression analysis suggests that irrespective of confounders, an increase in albumin of 1 g/dL reduces the likelihood of CKD by 27%. Likewise, advanced CKD (eGFR < 30) increases the likelihood of malnutrition (albumin < 3,5 g/dL) more than three times (five times after adjusting for comorbidities and country). This data confirms the strong relationship between eGFR and albumin levels.

One of the most important findings was the poorer MNA tests results in CKD participants. Generally, the studied group of older people was relatively fit and with good nutritional status. According to MNA score < 24 a risk of malnutrition was observed in only 338 subjects (15.7%). Nevertheless, this risk increased together with reduced kidney function. Positive correlation for both genders and also statistically lower MNA result in the group with eGFR 15–30 indicates an increasing risk of malnutrition in CKD subjects. In contrast, the MNA SF screening test did not significantly correlate with eGFR nor differ between eGFR stages, which may suggest limited application of MNA SF as malnutrition screening tool in older adults 75+ [56].

Overall, findings from the present study may be relevant to clinicians dealing with older patients. The assessment of anthropometric measures and comorbidities may help to identify patients at risk of negative cardio-metabolic and renal outcomes to be referred to specific care pathways aimed at reducing the burden of abdominal obesity and other risk factors. On the other hand, the identification of patients at risk of malnutrition is equally relevant when considering the consequences of malnutrition in terms of frailty, functional decline, sarcopenia, falls, hospitalization and death. In both cases, regular and careful monitoring of nutritional status using validated instruments would be needed.

Limitations of our study deserve to be mentioned. The cross-sectional design limits the interpretation of the observed associations, and the analysis of currently ongoing prospective phase of the SCOPE study may reveal different results. The population in the initial study included people with no advanced chronic kidney disease, and therefore inferences may relate to risk factors rather than symptoms in the final stages of CKD. We did not analyze the impact of diet on our findings. The observation that excessive body weight, and especially visceral obesity is associated with CKD, whereas malnutrition may appear in advanced stage of chronic kidney disease in both sexes suggests the need for a personalized diet and appropriately selected physical activity to prevent both malnutrition and obesity [3]. However, 24 h-dietary recall data will be available within the SCOPE study and warrants further analyses in this very interesting topic. Body composition, as well as steroid hormones and hypothalamic-pituitary-adrenal/gonadix axis were not included in the present study. Nevertheless, both are planned to be included in future studies within the SCOPE project.

As for strength, we studied a real world population of older people aged 75 or more enrolled without stringent exclusion criteria who underwent a comprehensive assessment of anthropometric and nutritional variables measured by standardized methods.

## Conclusions

The population of community dwelling 75+ with CKD shows generally features of overweight and obesity with small prevalence of malnutrition. For anthropometric measures, the strongest association with eGFR and the highest odds of CKD were identified using WC, HC, AC, CC and WHtR. Albumin level and MNA, but not MNA SF, indicated an increasing odds of malnutrition together with decreasing eGRF.

## Data Availability

Data will be available for SCOPE researchers through the project website (www.scopeproject.eu).

## Abbreviations

AC: Middle arm circumference
BMI: Body mass index
CAD: Coronary artery disease
CC: Calf circumference
CIRS-G: Cumulative Illness Rating Score for Geriatrics
CKD: Chronic kidney disease
COPD: Chronic obstructive pulmonary disease
CVD: Cerebro Vascular Disease
eGFR: Estimated glomerular filtration rate
ESRD: End stage renal disease
HC: Hip circumference
HF: Heart failure
MNA SF: Short Form of Mini Nutrition Assessment
MNA: Mini Nutrition Assessment
SCOPE: Screening for Chronic Kidney Disease Among Older People Across Europe
WC: Waist circumference
WHR: Waist-to-Hip ratio
WHtR: Waist-to-Height ratio
